# Contrasting Views of the Electric Double Layer in Electrochemical CO_2_ Reduction: Continuum Models vs Molecular Dynamics

**DOI:** 10.1021/acs.jpcc.4c03469

**Published:** 2024-06-14

**Authors:** Evan Johnson, Sophia Haussener

**Affiliations:** Laboratory of Renewable Energy Science and Engineering, École Polytechnique Fédérale de Lausanne, Station 9, 1015 Lausanne, Switzerland

## Abstract

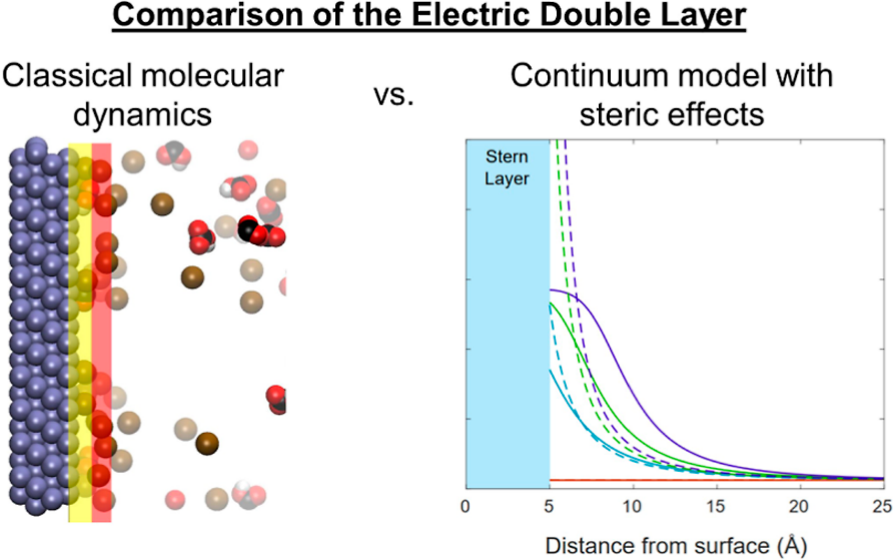

In the field of electrochemical CO_2_ reduction, both continuum models and molecular dynamics (MD) models have been used to understand the electric double layer (EDL). MD often focuses on the region within a few nm of the electrode, while continuum models can span up to the device level (cm). Still, both methods model the EDL, and for a cohesive picture of the CO_2_ electrolysis system, the two methods should agree in the regions where they overlap length scales. To this end, we make a direct comparison between state-of-the-art continuum models and classical MD simulations under the conditions of CO_2_ reduction on a Ag electrode. For continuum modeling, this includes the Poisson–Nernst–Planck formulation with steric (finite ion size) effects, and in MD the electrode is modeled with the constant potential method. The comparison yields numerous differences between the two modeling methods. MD shows cations forming two adsorbed layers, including a fully hydrated outer layer and a partial hydration layer closer to the electrode surface. The strength of the inner adsorbed layer increases with cation size (Li^+^ < Na^+^ < K^+^ < Cs^+^) and with more negative applied potentials. Continuum models that include steric effects predict CO_2_ to be mostly excluded within 1 nm of the cathode due to tightly packed cations, yet we find little evidence to support these predictions from the MD results. In fact, MD shows that the concentration of CO_2_ increases within a few Å of the cathode surface due to interactions with the Ag electrode, a factor not included in continuum models. The EDL capacitance is computed from the MD results, showing values in the range of 7–9 μF cm^–2^, irrespective of the electrolyte concentration, cation identity, or applied potential. The direct comparison between the two modeling methods is meant to show the areas of agreement and disagreement between the two views of the EDL, so as to improve and better align these models.

## 1 Introduction

Employing electrochemical reduction (CO_2_R) to convert carbon dioxide (CO_2_) into valuable chemicals and fuels is a pathway for reducing greenhouse gas emissions and promoting a sustainable chemical industry. Various modeling approaches have been used to understand the underlying physics governing these catalytic reactions, with the eventual goal of fostering conditions that will maximize the production rate and selectivity of the desired product.

Among the modeling methods employed, continuum models solve for species transport in the electrolyzer device and components, which are applicable above the molecular scale, typically in the nm to mm range. These models have been used to study catalyst morphology,^1^ parasitic reactions,^2^ and gas solubility effects.^2,3^ In recent years, continuum models have been modified in an attempt to more accurately model the electric double layer (EDL), with a term added to include steric (ion size) effects. The steric term considered here, applied to the Poisson–Nernst–Planck (PNP) formulation, was derived by Wang^4^ and later applied to CO_2_R by several authors.^5−9^ Others have pursued ionic-size terms with the Poisson–Boltzmann formulation.^10^ In our recent work, we focus on the Stern layer portion of the model and the surface charge boundary condition.^11^ However, definitive experimental evidence of steric effects in CO_2_R is hard to obtain due to the multitude of interacting phenomena occurring during the catalytic reaction, with cations both facilitating the CO_2_ reaction^12^ and—according to models incorporating steric exclusion—hindering CO_2_ transport to the reaction plane.^5,11^

Classical molecular dynamics (MD) has been applied to many fields, but it has been rarely used to study the nature of the EDL in CO_2_R. In one notable example, Buckley et al.^13,14^ investigated the nature of the electrode–electrolyte interface on Ag and Cu catalysts, with a cation modifier (quaternary ammonium) material added to the catalyst surface. Only very moderate applied charges were investigated, with little analysis of the local electrical potentials or species concentrations near the EDL.

Fortunately, electrostatics has been a focus in the closely related field of (super)capacitors, where MD methods to model electrodes at a constant electrical potential have been recently developed.^15−17^ In the constant potential method (CPM), the charge of each electrode atom is found in an energy minimization scheme to achieve the specified potential of the electrode. This treatment is more physically realistic for a conductive metal electrode than the simpler (and often used, historically) alternative, which is to assign a small fixed charge to each electrode atom, termed the fixed charge method (FCM). The CPM allows the electrode to dynamically interact electrostatically with the approaching charged particles, an effect neglected by the FCM. For example, when a cation approaches an electrode, the presence of the cation induces an extra negative charge locally in the electrode, which attracts the positively charged particle even more. This is often called the image charge or induced polarization. Thus, FCM (which neglects induced polarization) underestimates the attraction of a charged particle to the electrode compared to CPM.^18^ CPM has been applied to model capacitors with ionic liquids,^17,19^ but numerous studies of the EDL have also neglected these effects, opting for the FCM instead,^20,21^ even while noting the known shortcoming of the method.

Forming and breaking of chemical bonds is more suited for ab initio molecular dynamics (AIMD) than classical MD, with studies in CO_2_R focusing more on transition states and reaction mechanisms than the EDL structure.^22,23^ Ions are slow moving compared to water and have large hydration shells, making it a challenge to accurately sample the whole EDL with AIMD.^10^ The central limitation of AIMD is its high computational cost, which limits the size and duration of simulations (e.g., to less than 1000 atoms and 100 ps). Because classical MD relies on empirically derived distance–potential relations to compute forces between atoms, usually parametrized from experiments or from AIMD, it carries a much lower computational cost than AIMD. This makes larger systems (>100,000 atoms) and longer durations (>10 ns) possible in MD, which are in the necessary length and time scales to capture the effects of the EDL with reasonably detailed time averages.

The aim of this study is to present a clear, direct comparison between the nature of the EDL as predicted by continuum models and by classical MD under CO_2_R conditions. Ideally, these models should agree with each other to present a comprehensive view of the EDL at multiple length scales. We present these EDL models side-by-side to bridge knowledge gaps that may be lacking, as researchers or research groups often focus solely on one modeling method or the other. We do not proceed to modify the two models to achieve an agreement at this point, but we present the comparison to lay the groundwork for such research.

## 2 Continuum Models

The “Gouy–Chapman–Stern” (GCS) description of the EDL consists of several individual layers and is presented in texts^24,25^ as well as in CO_2_R modeling research.^5^ We refer to the Stern layer or Helmholtz layer as the region between the metal electrode and the outer Helmholtz plane (OHP), the plane of closest approach of hydrated, nonspecifically adsorbed cations. The continuum treatment of the Stern layer uses a surface charge boundary condition and has been discussed in detail previously.^11^ The inner Helmholtz plane (IHP) passes through the center of any specifically adsorbed ions,^24^ but in continuum CO_2_R modeling, the common assumption is that no ions are specifically adsorbed, so the IHP is not used in these models. Outside of the OHP is the diffuse layer, where electroneutrality is not obeyed (there is charge separation), with a layer thickness of ∼3 nm.^11^ Beyond that lies the diffusion layer, where there are concentration gradients and electroneutrality is obeyed until the bulk is reached at ∼100 μm, where there are no longer concentration gradients. This view of the EDL is certainly simplified compared to reality, where Faradaic currents, 2D and 3D effects, and molecular-level interactions may play a role. However, it is still a useful description for understanding and visualizing the physics governing different parts of the EDL.

Various continuum equations have been used in electrochemical models. In its original form, the Poisson–Boltzmann (PB) formulation does not account for finite ion size (steric) effects. Steric effects were originally added by Bikerman^26^ and have since been refined by numerous researchers. Unlike PB, the PNP formulation can model how systems change with time, and researchers have added steric effects to the PNP equations as well, such as Kilic et al.^27^ whose model is for symmetric electrolytes. In this study, we use the generalized modified PNP (GMPNP) model by Wang et al.,^4^ which has the further advantages of working with asymmetric electrolytes and multiple ionic species, and it has been previously used in CO_2_R.^2,5,6,8,11,23^ This formulation is given by eqs 1–3.  is the molar flux of species *i*, *C*_*i*_ is the concentration, *D*_*i*_ is the diffusivity, *z*_*i*_ is the charge, *F* is Faraday’s constant, *R* is the universal gas constant, and *T* is the temperature. The simplest of the continuum formulations commonly employed for CO_2_R is the reaction–diffusion equation, where species transport is only governed by diffusion, and a source/sink term accounts for the homogeneous reactions (first term in eq 2 and *R*_*i*_ in eq 1). If electrostatic migration of ions in an electric field is modeled, this is accounted for with the second flux term in eq 2, and coupling to the Poisson equation for electrical potential is required (eq 3). This forms the PNP set of equations, where ϕ is the electrical potential vs the potential of zero charge (PZC), ε_0_ is the permittivity of free space, ε_*e*_ is the relative permittivity of the electrolyte, and ρ_*f*_ is the *free* charge density from the imbalance of ions . It is important to note that the charge density in eq 3 is only the free charges (i.e., ions), as bound charges (i.e., water molecules) have been accounted for in the derivation of this version of the Poisson equation using the linear dielectric assumption and the relative permittivity.^11,28^

Because the original PNP formulation does not consider the finite size of ions, at extreme potentials ions attracted to the electrode can reach unphysically high levels (e.g., 21 M or higher).^5^ This prompted the development of a steric size term (eq 2, third term), forming the GMPNP formulation,^4^ where *N*_A_ is Avogadro’s number, and *a*_*j*_ is the hydrated diameter of species *j*. While the GMPNP formulation certainly limits the cation concentration at the (negatively charged) cathode to a more reasonable level than PNP, it has not yet been validated experimentally for CO_2_R or with atomistic models, which is a central aim of the present study. However, as noted in our recent article,^11^ the PNP model computes cation concentrations only slightly above the steric limit if a reasonable Stern layer capacitance (e.g., 20–25 μF cm^–2^) is used, as opposed to studies where higher values (100–200 μF cm^–2^) have been used.^5,8,9,29,30^ Apart from enforcing a limit on the cation concentration, the GMPNP model also predicts that other species, including CO_2_, will be crowded out due to steric effects, reducing their concentrations within a few nm of the electrode surface, whereas the original PNP model incorporates no such exclusion.
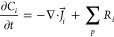
1
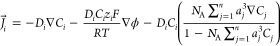
2
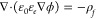
3

Equations 1–3 model the species transport in the diffuse and diffusion layers (between the OHP and the bulk). In the Stern layer, between the metal electrode and the OHP, no ions are present under the assumption of no specific ion adsorption. This makes the potential profile linear across the Stern layer, according to Poisson’s equation. Equation 4 is the boundary condition often used, which accounts for this linear potential drop, where *x*_Stern_ and ϵ_Stern_ are the thickness and permittivity of the Stern layer. As discussed in detail,^11^ the relative permittivity is not the same in the Stern layer as in the free electrolyte, and we recommend substituting a Stern layer capacitance, *C*_Stern_ for . Experimentally found values of *C*_Stern_ are often reported in the range of 20 to 25 μF cm^–2^ at potentials well below (e.g., 0.5 V below) the PZC.^11,31,32^ In this study, to visualize the potential profile across the Stern layer, we specify *x*_Stern_ = 5 Å and ϵ_Stern_ = 11.3, resulting in *C*_Stern_ = 20 μF cm^–2^.
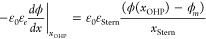
4

Equations 1–3 are solved under 1D, steady-state conditions, for a domain extending from the electrode surface to the bulk electrolyte at a distance of 100 μm. Equations are solved with the finite element method using COMSOL v6.0.^33^ The species *i* modeled include CO_2_, HCO_3_^–^, and the cation (Li^+^, Na^+^, K^+^ or Cs^+^), with bulk electrolyte concentrations specified as Dirichlet boundary conditions. Since the CO_2_ to CO electrochemical reaction is not modeled in MD (due to the exceptionally high computational requirements), the continuum model is also run under the condition of no electrical current or electrochemical reaction. Thus, a zero-flux boundary condition is used for each species at the electrode. Equation 4 is the boundary condition for the Poisson equation, and properties are found in Table S1.

## 3 Molecular Dynamics Model

In MD models, the forces between each atom and its neighbors are computed at each time step, and a time integration of Newton’s second law is then performed to calculate the new atom positions after a duration of one time step. The van der Waals force between each pair of atoms is modeled with a distance–potential relation (a “force field”), with a common choice being the 12–6 Lennard-Jones (LJ) potential,^34^eq 5. σ is the distance at which there is zero potential, ε is the depth of the potential well, and *r* is the radial distance between the two particle centers. The LJ potential is neglected for atoms above a cutoff distance of *r*_c_LJ__. The Coulombic potential (*E*_C_) between atoms *i* and *j* is calculated with eq 6, where *q*_*i*_ and *q*_*j*_ are the atomic charges, for atom pairs within a distance of *r*_c_C__. However, long-range Coulombic interactions for atoms at a distance greater than *r*_c_C__ are not negligible, and they are accounted for using a Fourier transform method (particle–particle particle–mesh^34^).
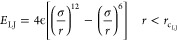
5
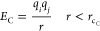
6

MD simulations are run in the open-source code LAMMPS (large-scale atomic/molecular massively parallel simulator)^34^ to simulate an electrolyte saturated with CO_2_. The “simple point charge extended” (SPC/E) water molecule^35^ is used, as it is one of the most commonly used water molecule models. The base case modeled is a 0.25 M KHCO_3_ electrolyte, and in later cases the cation is changed to Li^+^, Na^+^, and Cs^+^. Parameters σ and ϵ are taken from literature, given in Table 1, and all molecules are considered rigid. OH^–^, H^+^, and CO_3_^2–^ are not modeled, as their bulk concentrations are several orders of magnitude lower than CO_2_, K^+^, and HCO_3_^–^. The LJ parameters are taken from different studies, so the geometric mixing rule is used for interactions between species. Results shown use the K^+^ parameters from Jiang,^20^ but we run a comparison using parameters from Dang^36^ as well, with almost no difference shown between the two (see Figure S3 for details). We use Lee’s parameters^37^ for Li^+^, and we note that Lee’s parameters for K^+^, Na^+^, and Cs^+^ are nearly the same as those from Dang. The TraPPE^38^ CO_2_ force field parameters are used in this study, and as discussed in more detail in Section 4.1, our results are consistent with numerous MD studies published with a variety of both CO_2_ and H_2_O molecules. These cross-checks show that the choice of model parameters among the published values does not have a large impact on the overall results or broad conclusions in this study. Still, a more thorough parametrization using DFT could be an area for future improvement.

**Table 1:** Lennard-Jones Parameters and Partial Charges Used in MD Simulations

atom	ϵ (kcal mol^–1^)	σ (Å)	mass (g mol^–1^)	partial charge (e^–^)	ref.
O (H_2_O)	0.1553	3.166	15.9994	–0.8476	(35)
H (H_2_O)	0	0	1.008	+0.4238	(35)
C (CO_2_)	0.053649	2.8	12.0107	+0.7	(38)
O (CO_2_)	0.1569891	3.05	15.9994	–0.35	(38)
H (HCO_3_^–^)	0	0	1.008	+0.4	(39)
C (HCO_3_^–^)	0.05763	2.785	12.0107	1.123	(39)
O #1 (HCO_3_^–^)	0.15539	3.1656	15.9994	–0.8338	(39)
O #2 (HCO_3_^–^)	0.15539	3.1656	15.9994	–0.8985	(39)
O #3 (HCO_3_–, bonded to H)	0.15539	3.1656	15.9994	–0.7907	(39)
Li^+^	0.16013	2.337	6.941	+1	(37)
Na^+^	0.1000	2.584	22.9898	+1	(36)
K^+^	0.08694	3.143	39.0983	+1	(20)
Cs^+^	0.1000	3.884	132.9055	+1	(36)
Ag	4.56	2.6326	107.8682	set by CPM	(40)

**Figure 1:**
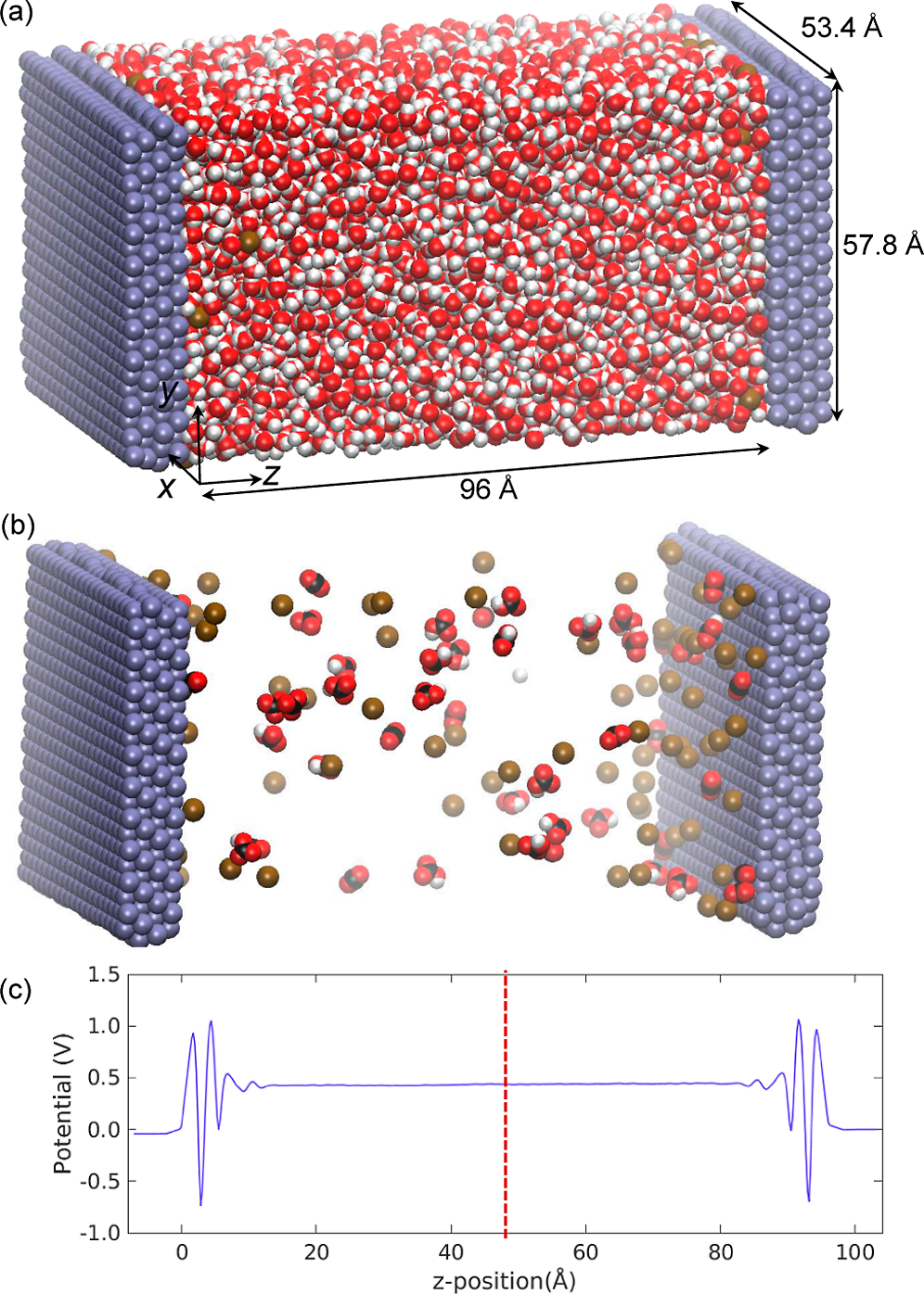
Modeled domain comprising two identical electrodes, for a 0.25 M KHCO_3_ electrolyte, with periodic boundaries in the *x* and *y* directions. Setup shown (a) with water molecules, (b) with transparent water molecules, and (c) potential profile calculated from atom charges, with data to be mirrored around the dashed red center line. Atom colors: C = black, O = red, H = white, K^+^ = brown, Ag = gray.

The modeled domain consists of two opposing electrodes with liquid electrolyte between them, as shown in Figure 1. The setup is visualized both with and without water molecules to reveal the dissolved species. The (111) face of an FCC lattice for Ag is modeled, with a lattice constant of 4.0868 Å.^40^ The electrode has dimensions of 53.4 by 57.8 Å (8 × 10 lattices), large enough to provide sufficiently refined averages over a 10 ns production run. Each electrode has a thickness of four Ag layers, thick enough that adding any more layers would not affect the results, as Ag atoms further from the surface carry no charge (see Section 4.1). In the setup modeled, both electrodes are negatively charged cathodes, making the setup symmetrical, with the two halves being under identical conditions. This symmetric setup is chosen because it avoids modeling the opposing positively charged electrode, which is not of interest for CO_2_R, thus reducing the computational cost by half. A similar setup has been used previously by Jiang et al.^20^ to study graphene capacitors. The electrodes are spaced 96 Å apart, far enough that the two opposing EDLs do not overlap and are essentially independent, as the spacing between the electrodes is much greater than two Debye lengths (6.2 Å each). Thus, the symmetric setup modeled with two negative electrodes is expected to behave the same as the negative side of a setup with one negative and one positive electrode. This comparison is shown in Figure S2, and indeed the results appear nearly identical.

In the simulations presented, a variant of the CPM is used, where the total charge on each electrode is specified at the outset instead of the potential.^41^ It is still a type of CPM simulation, with individual electrode atom charges varying in an energy minimization algorithm (not to be confused with the FCM where individual atoms are assigned a fixed charge throughout the simulation). Tee and Searles^42^ refer to this charge-specified variant of the CPM as ConQ to differentiate it from the original constant-potential version, ConP. They show the two versions are thermodynamically identical, though the dynamics are faster to approach equilibrium with ConQ. Using ConQ allows us to specify the total electrode charge at the outset, and to the balanced electrolyte we add an identical number of extra cations to keep the simulation charge neutral, as required in periodic-boundary MD simulations. For example, 36 K^+^ and 36 HCO_3_^–^ are added originally to form a balanced electrolyte and then a total electrode charge of 20 e^–^ (10 e^–^ on each electrode) is specified along with an additional 20 K^+^. We use the ConQ version of CPM because in ConP the electrode charge is not known a priori, so it is not possible to balance the electrolyte and the electrode charges. These simulations were run using the recent CPM implementation in the package LAMMPS-ELECTRODE.^41^

The potential within the electrode and throughout the electrolyte is found in postprocessing. The right half of the simulation is mirrored around the center (red line in Figure 1c); then all charges are divided into layers based on the *z* location. The electric field is found at each *z*-position by solving eq 7, where Δ*z* is the layer thickness (taken as 1/500 of the domain length, ∼0.19 Å) and ρ_*t*_ is the *total* charge density (i.e., all ions and atoms with partial charges, including water molecules). The potential profile is then found with eq 8. In differential form, this is the Poisson equation, . However, it is important to distinguish between this Poisson equation and the one used in continuum modeling (eq 3), as they are related but not the same. This equation includes the *total* charge density, ρ_*t*_, which includes all charged atoms (i.e., including water, which is neutral overall but has charges on H and O atoms in MD), and it does not use the relative permittivity. Equation 3 is derived from this equation by assuming the electrolyte behaves as a linear dielectric material, and it accounts for the polarization of water in a continuum sense by using the relative permittivity, an experimentally measured material property. After substituting the relative permittivity into the differential Poisson equation, only the *free* charges (i.e., ions) are included in the charge density, ρ_*f*_, in 3.^11,28^
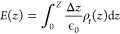
7
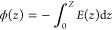
8

Simulations were run using an NVT ensemble, a Langevin thermostat, and a time step of 1 fs. The systems were allowed to equilibrate using simulated annealing,^21^ followed by a 10 ns production run over which time-averages were calculated. In each simulation, 36 HCO_3_^–^ and 36 cations were added to the domain, plus additional cations to balance the electrode charge. The result was a roughly 0.25 M electrolyte concentration. Twelve CO_2_ molecules were added to achieve slightly above 0.034 M concentration, the saturated concentration under standard conditions.^43^ With this setup, it is not possible to specify the exact bulk concentrations at the outset, as a discrete number of ions and molecules are added to the domain at the beginning of the simulation. Some of these will form the EDL and others will remain outside the EDL to form the bulk, with the resulting concentrations determined in post-processing. To adjust the bulk concentration, the simulation must be started over after adjusting the number of inserted particles. Such iterations were performed until the bulk concentrations approximately matched their target values of 0.25 M electrolyte and 0.034 M CO_2_.

Finally, it is important to note what classical MD can and cannot model. Classical MD can capture van der Waals forces and electrostatics, including dynamic image charges in the electrode with CPM. It cannot capture chemisorption where electrons are shared between the metal surface and the adsorbate. Since chemisorption of ions or water may play a role in the EDL structure, especially regarding the EDL capacitance,^44^ it would be useful to corroborate the trends shown using DFT/AIMD.

## 4 Results

Simulation results are obtained for electrode charges of 0, −10, −20, and −30 e^–^ on each electrode. These are referred to by the EDL charge, the positive charge values, throughout this section. For example, the results labeled with “*q* = 30” have a charge of −30 e^–^ on each electrode, and the electrolyte has an excess of 60 cations compared to anions. All simulations are run with the two-cathode configuration, with the results presented being an average from the two sides. In all figures, distances are measured from the electrode surface, where *z* = 0 is the center of the cathode atoms at the electrode–electrolyte interface. Section 4.1 focuses on the KHCO_3_ electrolyte, and Section 4.2 compares results with different cations. Section 4.3 investigates the coordination of cations with water and quantifies adsorbed layers as a surface density. Section 4.4 focuses on the CPM simulation, Section 4.5 focuses on steric effects, and Section 4.6 is on the EDL capacitance.

### 4.1 KHCO_3_ Electrolyte, Varying Cathode Charge

**Figure 2:**
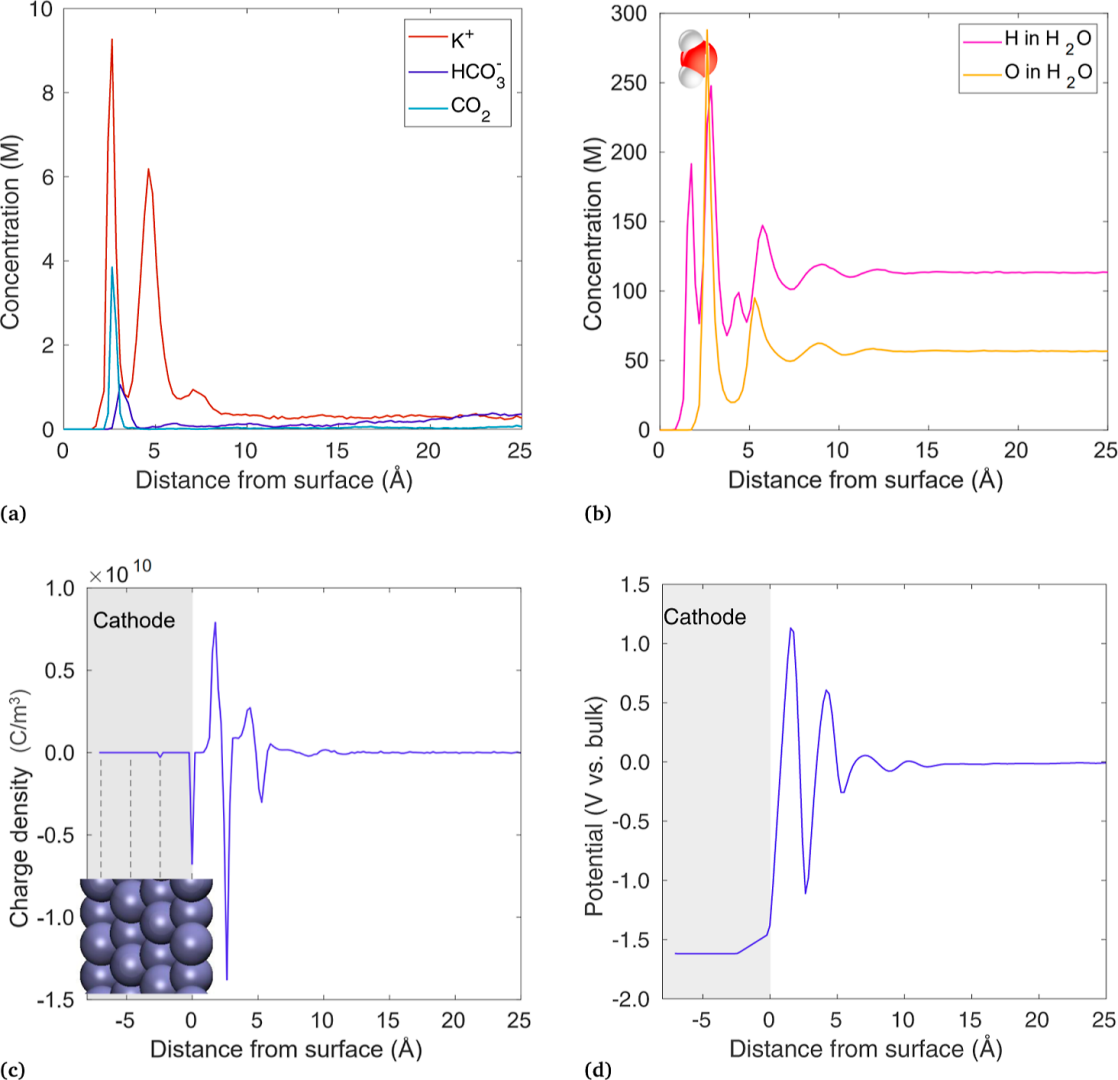
Time-averaged results for *q* = 30 e^–^ with 0.25 M KHCO_3_ electrolyte, showing (a) concentrations of K^+^, HCO_3_^–^, and CO_2_, (b) concentrations of O and H in H_2_O and a H_2_O molecule shown for scale, (c) charge density from all atoms, where the center of each Ag cathode layer is indicated by a dashed gray line, and (d) electrical potential.

MD results for a 0.25 M KHCO_3_ electrolyte with a charge of *q* = 30 e^–^ are shown in Figure 2, including concentrations of dissolved species, concentration of water atoms, charge density, and potential. Figure 2a shows the species concentrations, including K^+^, HCO_3_^–^, and CO_2_. Electrostatics attracts K^+^ to the cathode, which increases in concentration from 0.25 M outside the diffuse layer to nearly 10 M near the cathode. The double peak in cation concentration is seen to varying degrees in all of the simulations. We refer to these throughout this work as the inner (closest to the electrode) and outer adsorbed layers. However, these do not match the textbook definition of the inner and outer Helmholtz planes exactly (see Section 4.3). Due to electrostatic repulsion, HCO_3_^–^ decreases from 0.25 M outside the diffuse layer to near zero at the cathode surface, though there is also a small surface layer present as well. CO_2_ has a concentration outside the EDL of roughly 0.034 M, and it also shows a peak near the cathode. The trends in concentration are discussed in more detail later in this section.

Figure 2b shows the concentrations of H and O of the water molecules. In the SPC/E water model, H and O have fixed partial charges of 0.4238 and −0.8476 e^–^, respectively (due to electronegativity); so they have electrostatic interactions with the Ag electrode atoms in addition to van der Waals forces. The negatively charged electrode attracts H and repels O atoms, forcing water molecules to rotate until the H points predominantly toward the surface. This is seen as the hump in H closest to the cathode surface (1.75 Å), which is devoid of O. O peaks at 2.75 Å, where H also forms a second peak. This peak in H is lower than expected from the 2:1 ratio of H to O atoms, as the H atoms are found mostly in the first and third peaks. The H and O concentrations form a type of damped oscillation, but it is not simply an underdamped oscillation, as hydration shells and the cation adsorption also complicate the picture. Oscillations eventually dwindle by ∼12 Å.

The charge density is shown in Figure 2c, along with an image to show the size and spacing of the Ag electrode atoms for comparison, with the center of each layer indicated by the dashed gray line. The cathode region is shaded gray, with the solid–liquid interface at *z* = 0 taken as the center of the Ag atoms facing the electrolyte. The charge density has a contribution from every atom, as even the atoms in H_2_O, HCO_3_^–^, and CO_2_ have partial charges. Within the cathode, the CPM simulation finds that almost all (96%) of the −30 e^–^ is distributed in the surface layer of Ag atoms, a small fraction of the charge (4%) exists in the second layer, and the third and fourth layers have essentially no charge. This matches expectations from electrostatics, as the minimum energy state of a charged conductor has the charge resting on the electrode surface (e.g., Faraday cages). The deeper layers of cathode atoms could be omitted, but they are kept in place for demonstration of the CPM model. In the liquid region, the charge density starts positive, due to the charged H and K^+^ being attracted to the electrode, followed by a strong layer of negative charge due to the layer of predominantly O, and the oscillations die out in the bulk. Finally, the potential profile is shown in Figure 2d, which starts at −1.87 V vs the bulk electrolyte and oscillates from negative to positive several times until 12 Å, with the oscillations largely due to the polarized water molecules at the solid–liquid interface. Taken together, these four graphs reveal some of the key behaviors of the EDL, with the surface interactions of H_2_O causing the oscillating charge and potential profiles, which in turn affect the distribution of ions near the electrode surface.

**Figure 3:**
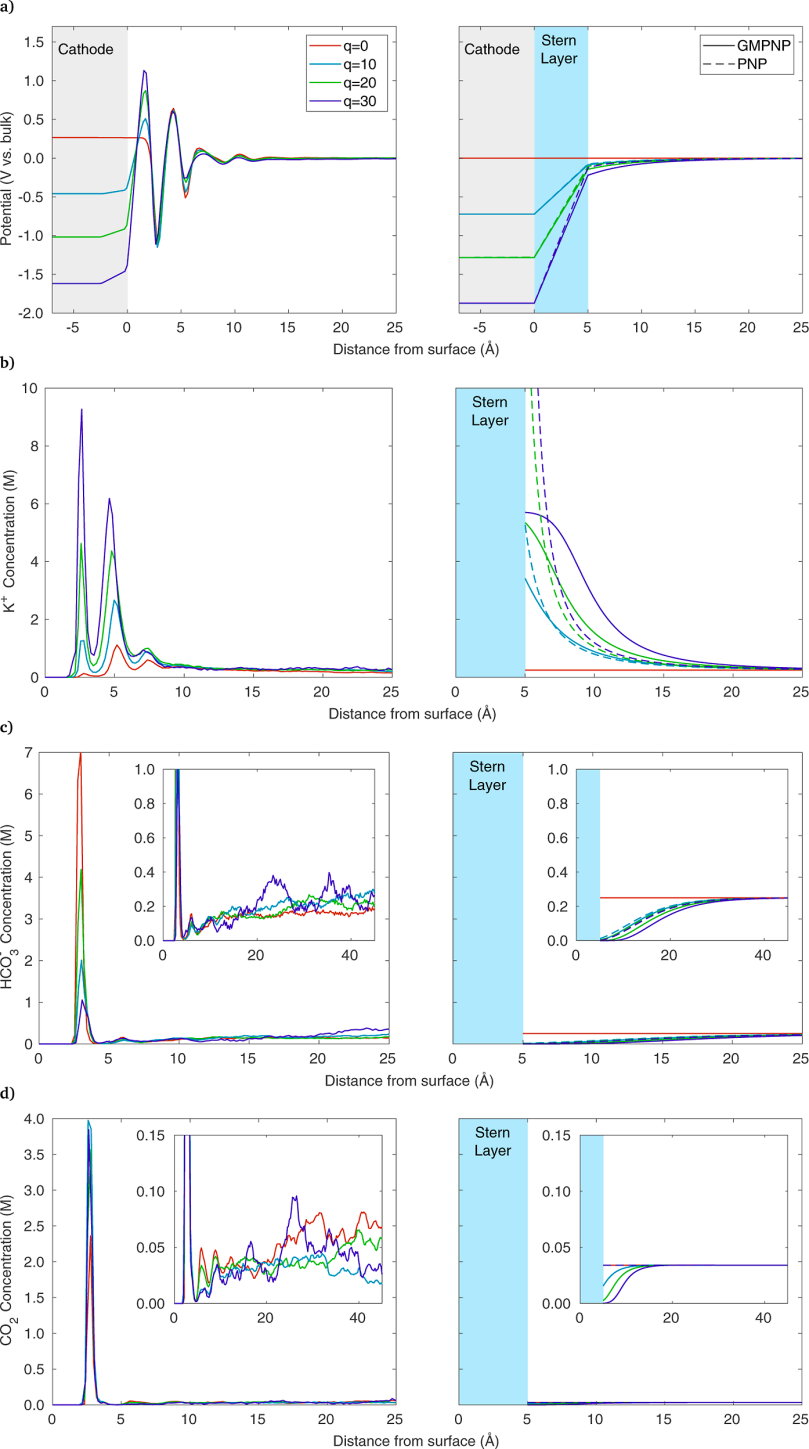
Comparison of results between MD (left column) and continuum simulations (right column), showing (a) potential vs bulk, and concentrations of (b) K^+^, (c) HCO_3_^–^, and (d) CO_2_. Distances measured from center of the Ag atoms at the electrode–electrolyte interface. Blue region represents a 5 Å thick stern layer, and cathode is shown in gray.

A direct comparison between MD and continuum results is shown in Figure 3, for KHCO_3_ and *q* = 0, 10, 20, and 30. MD results are shown in the left column and PNP/GMPNP results are shown on the right. The continuum simulations are run under conditions matching MD, with the bulk electrolyte concentration of 0.25 M KHCO_3_, a CO_2_ concentration of 0.034 M, and no current density (no CO_2_R reaction). A total thickness of 100 μm is used in the continuum model, but the diffusion length chosen has no impact because no products are generated. The Stern layer thickness and relative permittivity are 5 Å and 11.3, respectively, to achieve a Stern layer capacitance of 20 μF cm^–2^ in eq 4. All other parameters (diffusion coefficients, steric sizes, etc.) match Bohra et al.^5^ and are given in Table S1. The MD domain has 96 Å between the electrodes, or 48 Å after mirroring and averaging, but only the first 25 Å are shown to focus on the region near the electrode.

The potential profile for each applied charge is shown in Figure 3a, where potentials are with respect to the bulk. The electrode charges from 0 to −30 e^–^ result in potentials of +0.265, −0.459, −1.018, and −1.608 V vs bulk. By definition, PZC is the condition of the *q* = 0 simulation, so we take the electrode potential in the *q* = 0 case to be the PZC, making the four electrode potentials 0, −0.724, −1.284, and −1.873 vs PZC. Thus, the continuum model is run with these potentials vs PZC (ϕ_*m*_ in eq 4). For reference, we note that the PZC over (111) Ag is roughly −0.45 V vs SHE,^45^ so these can be considered −0.45, −1.174, −1.734, and −2.323 V vs SHE.

The first disagreement between the two modeling methods is visible in the PZC state, where MD shows the potential of the electrode is +0.265 V vs bulk, whereas in the continuum model, the electrode, electrolyte, and bulk potentials are all equal at the PZC. The MD model includes interactions between the electrode surface and the electrolyte molecules, including van der Waals forces and electrostatics between Ag and the partial charges on the water atoms. Note that an extra attractive force from image charges still exists within CPM when a charged particle approaches the electrode, even though the overall electrode charge remains zero. The result of these electrode–electrolyte interactions is that a slight positive potential must be applied to the electrode to drive away enough H such that the electrode charge remains zero, to achieve the PZC state. Such effects are not included in the continuum model, so the PZC and the bulk potentials are simply equal.

An obvious difference between the MD and continuum results is the oscillating nature of the MD potential profile, compared to the uniformly increasing potential in the continuum models. As discussed regarding eqs 1–3, the potential in PNP/GMPNP is modeled with Poisson’s equation after taking the polarization of water into account in a continuum sense by using the relative permittivity.^11,28^ This treatment means the oscillations in charge and potential caused by the polarized water layers are not spatially resolved. In the continuum model, the potential varies linearly across the Stern layer, as there are no ions between the electrode and the OHP under the assumption of no specific adsorption typically used in CO_2_R models. Since the potential within 12 Å of the electrode is quite different between the two modeling methods, it is unsurprising that the ionic concentrations found are also different, as shown in the following figures.

Figure 3b shows the concentration of K^+^, which peaks near the cathode in both modeling methods. A double peak is predicted by MD, indicating that some K^+^ ions are closely adsorbed (peak at 2.6 Å) and some rest further away (peak at 4.6 Å), with the concentration eventually decaying to the bulk value away from the electrode. The PNP and GMPNP models do not include the complex phenomena that result in these layers of adsorption, which include a combination of van der Waals forces, electrostatics (including image charges) between the electrode and electrolyte atoms, water molecules and their polarization, and cation hydration. Though the continuum models do not capture the double-peak behavior shown in MD, the GMPNP model results show the K^+^ concentrations in a similar range, eventually reaching the steric limit of 5.73 M. In contrast, the PNP model peaks at 36 M (above the region plotted) for the *q* = 30 case, which is likely too high to be realistic. The PZC case (*q* = 0) shows another difference between MD and the continuum models. With *q* = 0, in the continuum model there is no electrostatic migration because the potential equals the bulk potential throughout the domain, so all concentrations are simply equal to the bulk. In MD, the electrode surface interactions still cause deviations from the bulk values, even though the electrode has a net zero charge. Overall, it appears that the continuum models present a simplified picture of the EDL by neglecting many of these molecular length-scale phenomena within several Å of the surface, yet GMPNP can still provide a reasonable approximation of the cation concentrations near the electrode.

Figure 3c shows HCO_3_^–^, where the concentration decreases toward the cathode in both models due to electrostatic repulsion. However, at 3 Å from the cathode, MD shows an abrupt peak, something not expected from the continuum perspective, but as described above, there are numerous surface interactions modeled in MD that keep some HCO_3_^–^ ions along the surface, which for an anion may include the region of positive potential around *z* = 3–5 Å. Though the bicarbonate ion is negative overall, the H and C atoms carry positive atomic charges, which are attracted to the cathode and can even induce their own attractive image charges. This can be seen in the Supporting Information (Figure S1a), where the H in HCO_3_^–^ is oriented toward the electrode due to its positive partial charge. The peak diminishes as the electrode charge becomes more negative, as the electrostatic repulsion begins to outweigh surface interactions. Though unexpected from the continuum perspective, adsorbed anions have been thought to play a role in the EDL capacitance.^31^ Still, a more in-depth study with DFT would be useful in corroborating these trends.

Figure 3d shows the CO_2_ concentration, which also indicates a tendency to stay near the electrode surface, peaking at 2.8 Å. While not expected from the continuum model perspective, several other MD studies have shown a similar peak. From the field of CO_2_ geo-sequestration, Javanbakht et al.^46^ used TraPPE CO_2_ in TIP4P water over a quartz substrate, while Iglauer et al.^47^ used the EPM2 CO_2_ molecule and TIP4P water. These simulations use uncharged substrates, but both studies still show a spike in CO_2_ concentration at the solid–liquid interface, suggesting that a van der Waals interaction is responsible, not (only) electrostatics. In one of the few MD studies available from the field of CO_2_R, Buckley et al.^13^ use the ReaxFF force field (a model that allows for bond breaking/forming) to simulate CO_2_ in electrolyte over an Ag substrate, and even though potentials are low and the CPM is not employed, an abrupt spike in CO_2_ concentration is shown in their results as well. Given that all of these MD studies used different CO_2_ molecules, water molecules, and substrates, but they all show a similar peak in CO_2_ at the solid–liquid interface, the result does appear repeatable in MD. The trend also agrees with an AIMD study on CO_2_ adsorption over Pt electrodes, where the potential energy of adsorption is computed as a function of the CO_2_ distance from the electrode, with an energy minimum at 3.2–3.4 Å,^48^ slightly farther than the CO_2_ adsorption shown here (though electrodes are different metals). Thus, numerous atomistic simulations have predicted a CO_2_ concentration to increase along the cathode due to interactions with the surface. This is in stark contrast to the continuum models, where PNP predicts a flat concentration profile, and GMPNP predicts a very small concentration due to steric effects. The orientation of CO_2_ is generally parallel to the surface (see Figure S1b). Note that while the peak in CO_2_ is well-resolved, the rest of the EDL has such a low CO_2_ concentration that the time-average is rather poor, so this is addressed with a higher concentration simulation in Section 4.5.

### 4.2 Effect of Cation Identity

**Figure 4:**
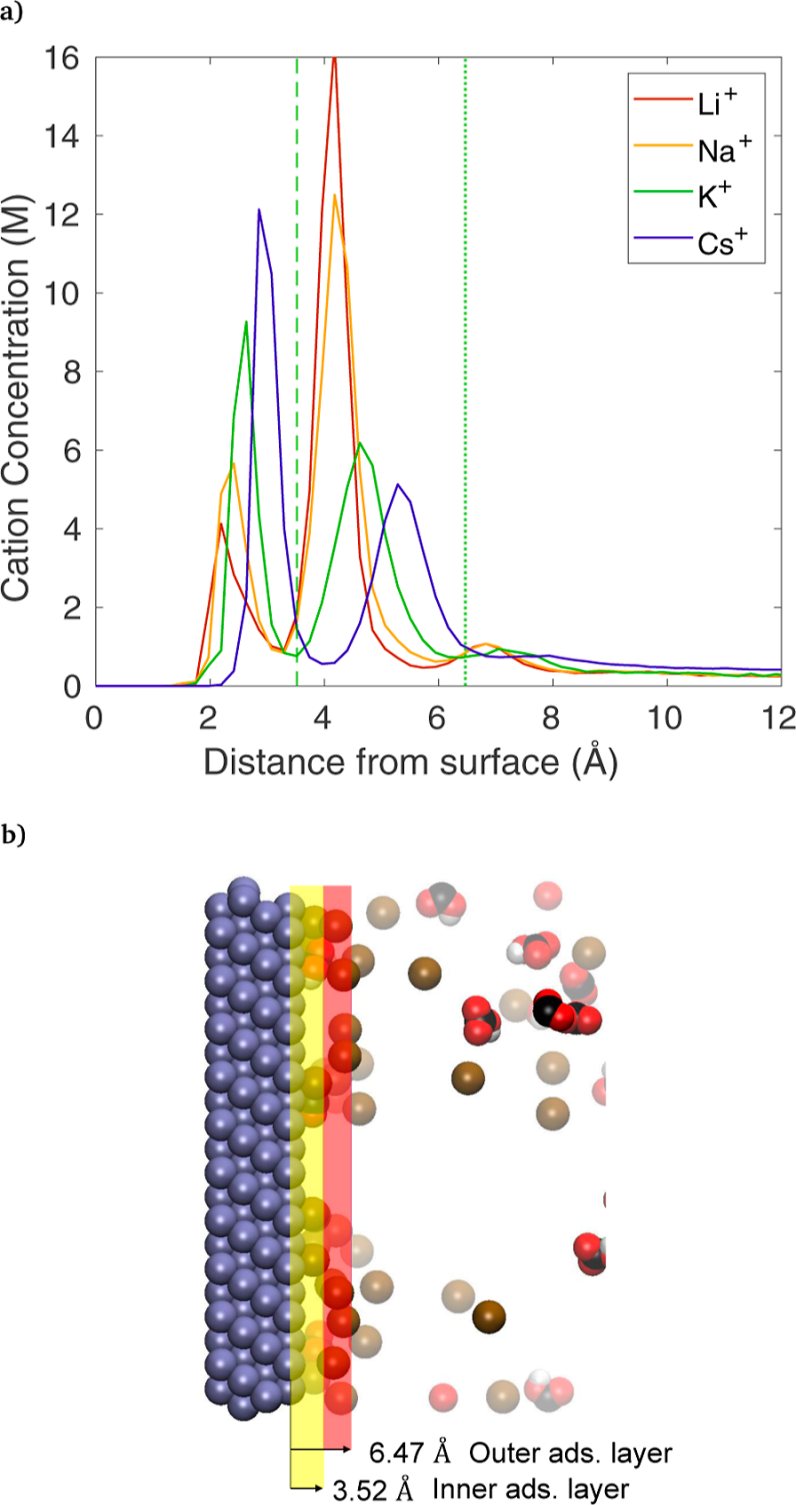
(a) Comparison of results from simulations with only the cation identity varied. Other conditions are identical to *q* = 30 in earlier figures. Vertical dashed and dotted lines represent the upper bounds of the inner and outer adsorbed K^+^ layers, respectively. (b) Snapshot of the KHCO_3_, *q* = 30 case, highlighting the inner and outer adsorption layers. Atom colors: brown = K^+^, red = O, white = H, black = C, gray = Ag, water molecules not shown.

Results are shown in Figure 4a for an identical simulation setup as in the previous section, with *q* = 30, but the K^+^ cation is changed to Li^+^, Na^+^, and Cs^+^. Larger cations show the strongest inner adsorbed layer, following the trend of non-hydrated ionic sizes (Cs^+^ > K^+^ > Na^+^ > Li^+^) matching the expectation that water is bound less tightly with large cations than with smaller cations. The outer layer shows the opposite trend, with a strong inner layer adsorption seemingly corresponding to a smaller outer layer adsorption. Unsurprisingly, the peak *z*-location of the inner adsorbed layer also matches the trend of ion size, with larger cations sitting further from the surface. In addition, a third layer is visible as well between 6 and 8 Å, but its concentration is very low, so it is not analyzed further. A snapshot of K^+^ with *q* = 30 is shown in Figure 4b, with each adsorbed layer highlighted. The span of each layer shown is defined by the local minima in Figure 4a. As expected from the cation concentration plot, a visual inspection shows that cations are grouped near the center of each layer, with relatively few situated with their center near the border. It is clear from watching the dynamic results that cations are not confined to one layer; they can be seen moving from the outer to the inner layer and back over the course of several picoseconds, but they do not linger in the border region.

Experiments show that CO_2_ is not reduced if there is no cation present,^12^ and the rate of CO_2_R depends highly on the cation identity, with Cs^+^ shown to enhance CO production over Li^+^.^12^ The different magnitude of the inner layer adsorption of Cs^+^ compared to Li^+^ may be part of the reason for this, either by fostering the local electrical field required for CO_2_R (possibly requiring bond bending^23^), by stabilizing intermediates,^12^ or by adding more reaction sites near cations. Answers to these questions may have to come from DFT/AIMD, but the tendency for larger cations to strongly adsorb would certainly affect the reaction region.

### 4.3 Surface Density and Cation Coordination

**Figure 5:**
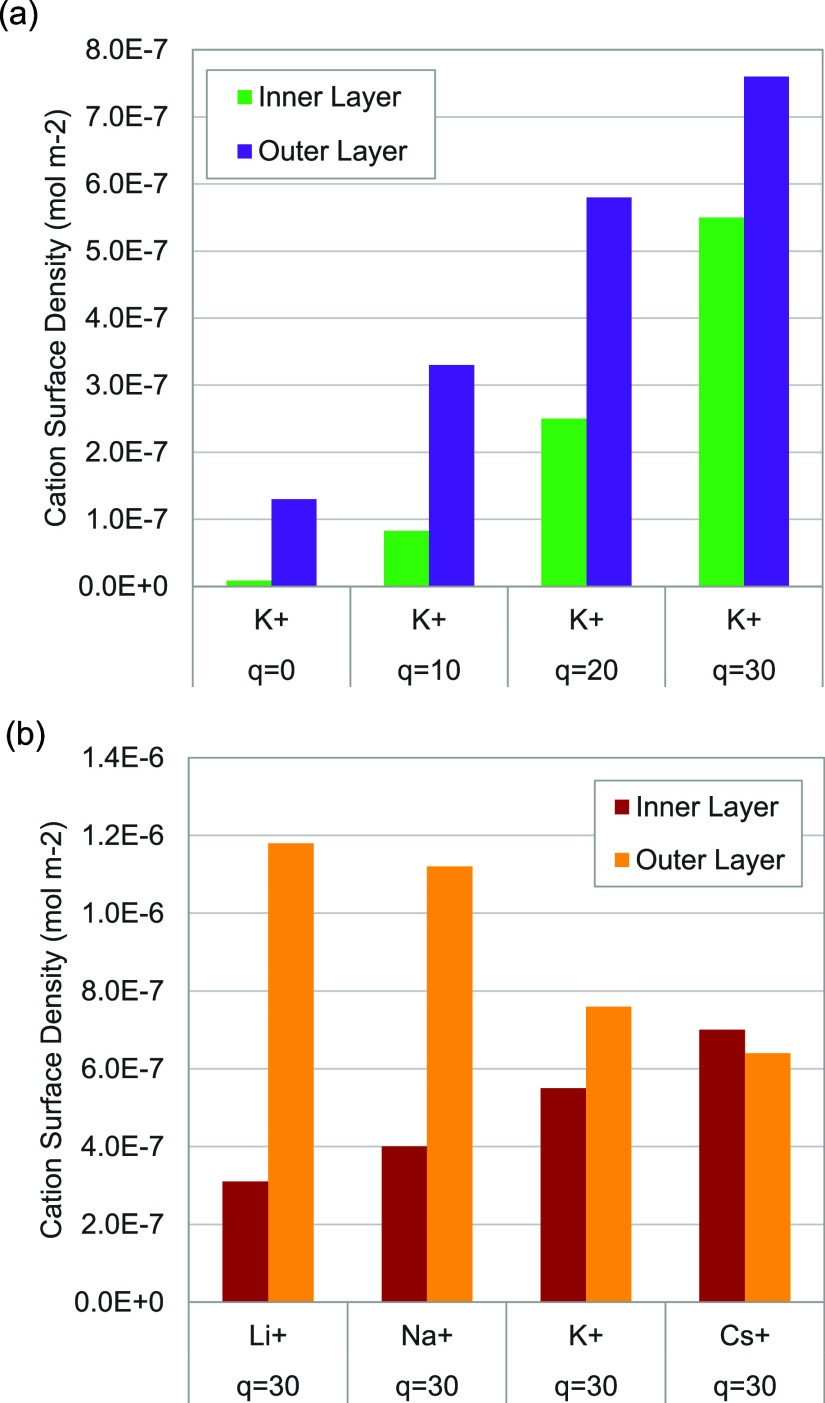
Surface density of cations for each adsorption layer, showing trends for (a) K^+^ with various electrode charges and (b) different cations with *q* = 30 e^–^.

Results for cation surface density and cation-H_2_O coordination number are given in Table 2. The two layers of cations are referred to as the inner and outer layers, with the upper boundary for each layer given in the first two columns of data, found from the minima in cation concentrations in Figure 4a. The next pair of columns gives the location of the peak cation concentration, which may be useful for the development of continuum models taking the position of these layers into account. Since these adsorbed layers are very thin, they can be interpreted as having surface densities as opposed to volumetric concentrations, which may be useful for microkinetic studies computing surface coverages. The surface densities are the integral under the cation concentration curves (Figures 3b and 4a) in each layer. To highlight these trends, Figure 5 plots these surface densities. As shown in Figure 5a, both the inner and outer layers increase in surface density at more negative electrode charges. As shown in Figure 5b, as the cation size increases, the inner layer is occupied more, and the outer layer is occupied less. In all cases except Cs^+^, the inner layer has a lower surface density than the outer layer, even though upon first inspection of Figure 3b it may appear that the opposite is true since the volumetric concentrations usually show a higher but thinner peak for the inner layer compared to the outer layer (e.g., K^+^, *q* = 30). Furthermore, the volumetric concentration calculated from such thin regions can be influenced by the choice of bin (slab) thickness. For example, in the extreme case of all cations lying on a plane, increasing the bin thickness by a factor of 2 reduces the calculated volumetric concentration by a factor of 2. In contrast, surface density avoids these artifacts, as it is the integral of all cations in the layer. This shows how the surface density (or coverage) becomes a useful metric on these length scales.

**Table 2:** Surface Density and Coordination Numbers of Cation to O in H_2_O for the Inner and Outer Adsorbed Layers^a^

simulation	upper boundary *z*-position (Å)		peak conc. *z*-position (Å)		cation surface density (mol m^–2^)		cation-H_2_O coord. num.		
	inner	outer	inner	outer	inner	outer	inner	outer	bulk
K^+^, *q* = 0	3.52	6.47	2.76	5.17	8.5 × 10^–9^	1.3 × 10^–7^			
K^+^, *q* = 10	3.52	6.47	2.78	4.97	8.3 × 10^–8^	3.3 × 10^–7^	5.7	7.6	7.3
K^+^, *q* = 20	3.52	6.47	2.60	4.79	2.5 × 10^–7^	5.8 × 10^–7^	5.7	7.7	7.3
K^+^, *q* = 30	3.52	6.47	2.64	4.62	5.5 × 10^–7^	7.6 × 10^–7^	5.8	7.7	7.2
Li^+^, *q* = 30	3.30	5.70	2.19	4.18	3.1 × 10^–7^	1.2 × 10^–6^	4.4	5.8	5.7
Na^+^, *q* = 30	3.30	5.95	2.42	4.18	4.0 × 10^–7^	1.1 × 10^–6^	4.6	6.1	5.9
Cs^+^, *q* = 30	3.96	6.83	2.86	5.29	7.0 × 10^–7^	6.4 × 10^–7^	8.2	10.1	10.1

a “Peak concentration” gives the location of the maximum cation concentration. The bulk region is considered as above 12 Å. The *q* = 0 case is not analyzed for surface density because there are essentially no cations in the inner layer.

**Figure 6:**
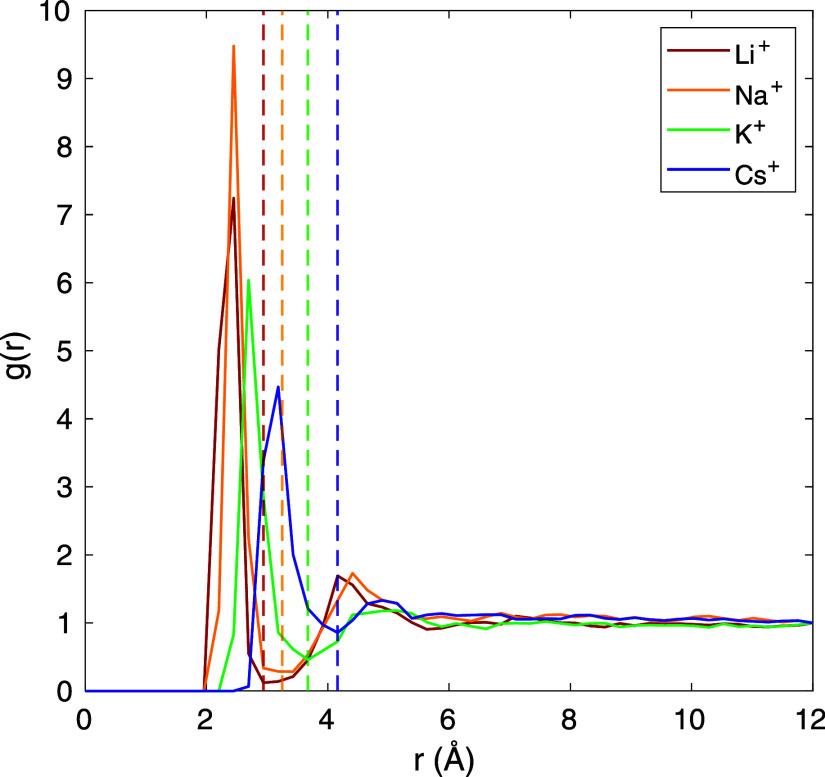
RDF, *g*(*r*), between cations in the first inner adsorbed layer and O in H_2_O, with *q* = 30, comparing cation identity. Dashed lines indicate the radial cutoff distance used to calculate the hydration shell, which increases with cation size.

Coordination numbers are used to quantify the hydration of cations. To find the coordination numbers, the radial distribution function (RDF) is first found between cations and O in H_2_O. RDFs are calculated separately for cations in the inner, outer, and bulk regions. The RDFs for cations in the inner layer are shown in Figure 6, for *q* = 30, comparing cation types. The edge of the hydration shell is taken as the minimum in the RDF curve and is indicated by the dashed lines, which follows the trend of cation sizes: 2.94 Å for Li^+^, 3.25 Å for Na^+^, 3.67 Å for K^+^, and 4.16 Å for Cs^+^ (which have a similar trend but are slightly smaller than those shown by MD simulations including other water molecules^49^). With the hydration shell cutoffs established, the coordination number is then calculated as the number of O in H_2_O with a radial distance less than this cutoff. As shown in the last three columns of Table 2, cations in the outer adsorbed layer are fully hydrated compared to the bulk region, with the small amount of excess hydration likely due to the presence of the H_2_O surrounding the inner layer. The inner layer has a consistently lower coordination number than the outer layer, showing that the hydration shell is partially shed when a cation moves from the outer to the inner layer. Comparing the results for K^+^ at different applied charges, it appears the hydration shell is essentially unaffected by changing the electrode charge, even though the number of adsorbed cations (surface density) increases substantially. Taking the trends in coordination number and surface density together, it appears that larger cations (Cs^+^) are coordinated with more water molecules (in both the bulk and adsorbed layers), as their shell has a larger radial cutoff distance. However, the shell is more easily shed than for smaller cations, leading to stronger inner layer adsorption.

In the EDL descriptions in texts,^24^ “specifically” adsorbed ions are said to have a chemical bond between the metal surface and the adsorbate. Likewise, Grahame defines specific adsorption as having a covalent bond, and it is specific to the ion type.^31^ Under this definition, the inner adsorbed layer discussed in the present study is not a specific adsorption, as no covalent bonding is modeled in MD. Trying to align the MD results with the GCS description of the double layer, it appears that the outer layer is the OHP, as the cations remain fully hydrated, making them “non-specifically” adsorbed. The MD results show the inner adsorbed layer being formed by cations moving from the outer layer toward the electrode, partially shedding their hydration shell for some time, before moving back to the outer or diffuse layers. Thus, the inner layer shown here does not fit neatly into the idealized and simplified GCS description of the EDL.

### 4.4 Constant Potential Method MD

All simulations discussed thus far are run using CPM, a method which is gaining in popularity for electrically conducting interfaces but is not yet widely implemented in MD studies. For example, Jiang et al.^20^ performed FCM simulations in a configuration similar to the present study, with aqueous solutions of K^+^, Rb^+^, Cs^+^, and Na^+^ cations, with Cl^–^ anions, and two opposing graphene electrodes. Their results show a strong inner adsorption layer only in the case of Cs^+^ and Rb^+^ and at the most negative electrode potentials. While the conditions are not identical (Jiang uses a graphene electrode), we find the same trends, with larger cations adsorbing more than smaller ones. However, our results show a stronger inner adsorbed layer, highlighting a key contribution of the CPM: neglecting image charges with FCM results in less ion attraction to the electrode.^18^

**Figure 7:**
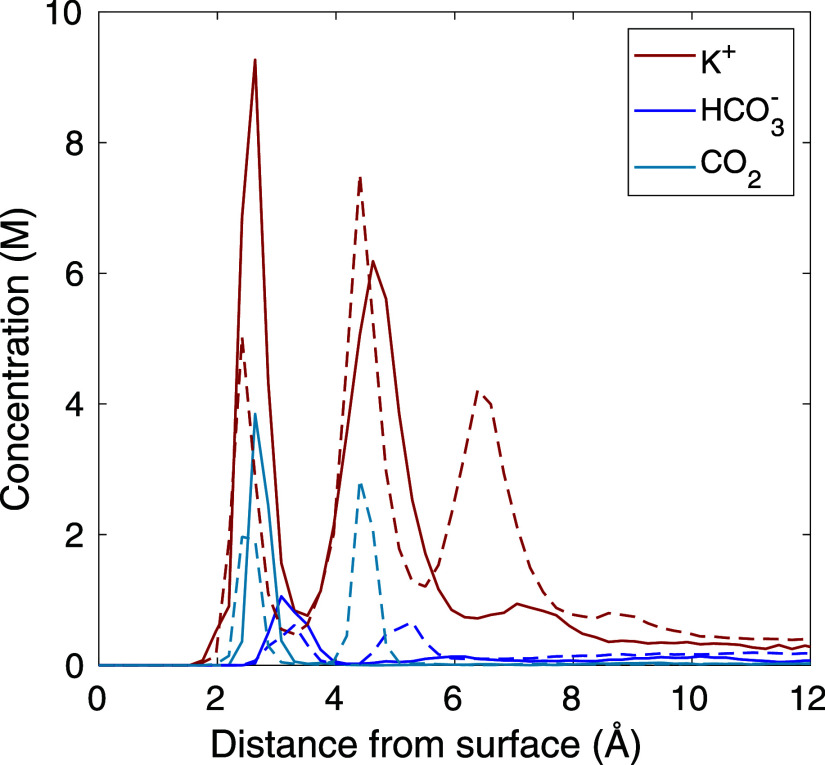
Species concentrations showing CPM (solid lines) and FCM (dashed lines) for *q* = 30 and KHCO_3_.

To show if FCM would suffice under our modeled conditions, Figure 7 shows results for the KHCO_3_, *q* = 30 simulation, calculated with both CPM (solid lines) and FCM (dashed lines). FCM shows a weaker inner adsorbed layer of cations, and in fact, FCM has a substantial third adsorbed layer not seen in any of the CPM simulations. The adsorbed layers of HCO_3_^–^ and CO_2_ also show a single peak near the electrode surface in CPM, whereas FCM predicts a lower peak at this location plus a second peak further into the electrolyte. Thus, using the FCM neglects some important physics and substantially changes the degree of adsorption in each of the species modeled. In the past, implementing CPM in MD may have been too onerous for many researchers (as expressed by Jiang et al.^20^), but as of May 2022, the LAMMPS-ELECTRODE package makes using CPM very straightforward. We found the computational cost of CPM to be ∼1.5 times that of FCM in these simulations.

### 4.5 Steric Effects

Steric effects, or the exclusion of species based on physical size, have been proposed to become relevant near the cathode surface, where cations accumulate at high concentrations due to electrostatic attraction.^4,5^ By including steric effects in continuum models, the GMPNP results show two main differences compared to the original PNP model. The first is a limit to the concentration of cations along the electrode surface to a level that respects their physical size. This is enforced by the denominator of the steric term in eq 2, which approaches zero when the concentration nears the steric limit, resulting in a large flux away from the electrode surface. The steric term is derived by assuming that cations behave as hard spheres with a diameter defined by their hydration shell, arranged in a simple cubic packing configuration along the electrode surface. We note that the simple cubic assumption is often not mentioned, and alternatively, a hexagonal close packing layer could have been chosen in the GMPNP derivation, which has a higher packing fraction and would raise the steric packing limit allowed by the GMPNP model. With the simple cubic packing assumption and a 6.62 Å hydrated ion diameter,^5^ the steric limit is 5.73 M for K^+^. The summation over “*j*” includes steric size contributions from all species, but under most conditions, the cation dominates the steric size summation near the cathode surface and the other species can be neglected. We note that PNP finds cation concentrations that are likely unrealistic (e.g., 34 M in Section 4.1), but in our previous work, we show that extreme cation concentrations have been exaggerated in previous PNP/GMPNP studies, as numerous researchers have used a Stern layer permittivity or capacitance far higher (e.g., 5–10 times higher) than is realistic.^11^ The second effect of GMPNP is that all other species, including CO_2_, are crowded out from the cathode surface. This can be seen in Figure 3d, where GMPNP predicts CO_2_ concentrations to decrease within about 12 Å of the cathode surface, and under the most negative electrode potentials, [CO_2_] is predicted to drop to near zero.

**Figure 8:**
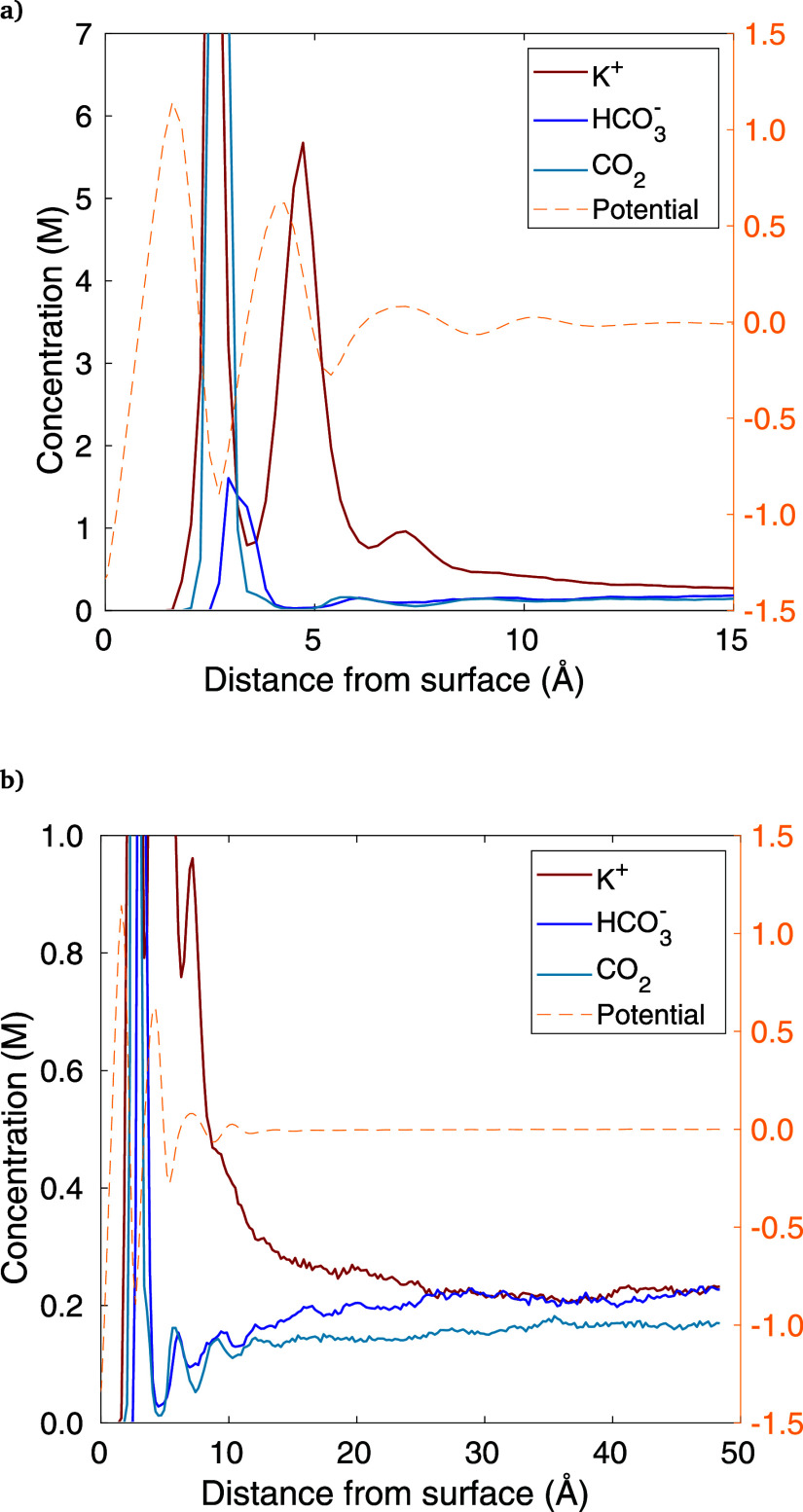
CO_2_ concentrations with 60 CO_2_ molecules modeled (5 times the base case), for KHCO_3_ and *q* = 30. The same data is shown in each chart, with (a) being a close-up of the adsorbed layers and (b) showing the entire width of the half-cell.

The CO_2_ concentrations in Figure 3d solved by MD are not well-resolved because the concentration is quite low, making it difficult to see any clear signs of steric CO_2_ exclusion. Therefore, an additional simulation is run to reveal any steric effects, by increasing the number of CO_2_ molecules from 12 to 60 and increasing the duration from 5 to 20 ns, with results shown in Figure 8. The CO_2_ concentration is higher than realistic at ambient pressure (though possible at elevated pressures), but the purpose is to show if steric effects can be seen in this exaggerated case. All else remains the same as the KHCO_3_ simulation, with *q* = 30.

In Figure 8, the same data set is plotted in (a) and (b), but they focus on different regions. Chart (a) is a close-up of the adsorbed layers, where CO_2_ peaks along the electrode surface. This CO_2_ peak directly coincides with the 12.5 M peak in K^+^, which is the opposite of the “crowding out” expected from the steric effect perspective. Thus, for the inner adsorbed layer, it appears that any steric effects, if present, are dwarfed by the surface interactions between CO_2_ and the electrode. In contrast, at the outer layer, there is a *dip* in CO_2_ that coincides with the K^+^ peak, which is minor but may indeed be a steric effect. Likewise, there is even a slight dip in CO_2_ coinciding with the third K^+^ peak. The potential is plotted alongside the concentrations to show any interactions between polarized water layers, ions, and CO_2_, but CO_2_ does not appear to have any correlation to the potential, as generally expected from a neutral molecule. Figure 8b shows how CO_2_ varies across the domain, where the concentration is essentially uniform further than 12 Å from the surface, similar to the continuum prediction. In summary, though there may be some mild steric effects occurring in the outer adsorbed layer(s), the dramatic drop in CO_2_ concentration predicted by GMPNP is not seen in the MD results. In fact, the MD model shows the opposite, with CO_2_ peaking along the electrode–electrolyte interface due to surface interactions. The difference in results between GMPNP and MD may also be because there are actually two adsorbed layers in the MD results instead of one in the PNP/GMPNP models, giving more space to each layer and leaving enough room for the hydrated cations, so CO_2_ is not crowded out.

### 4.6 Double-Layer Capacitance

Our understanding of the EDL and the models used to describe it are still evolving. While the present work is focused on the structure of the EDL, experimental work often focuses on EDL capacitance, since it is measurable with either electrochemical impedance spectroscopy or cyclic voltammogram data. GCS theory assumes that the EDL comprises an inner layer and a diffuse layer behaving as capacitances in series. This implies that the diffuse layer capacitance dominates in dilute solutions, while the inner layer capacitance dominates at high concentrations.^31^ GCS theory predicts a minimum in the double-layer capacitance at the PZC. As shown by Grahame,^31^ Hg electrodes with non-adsorbing anions follow these trends, but systems relevant for electrochemistry such as Ag and Pt often do not follow this ideal behavior. Though it is fundamental to electrochemistry, it is still unclear what causes these large deviations from GCS theory, and understanding this behavior is an active field of research.^44,50−52^

**Figure 9:**
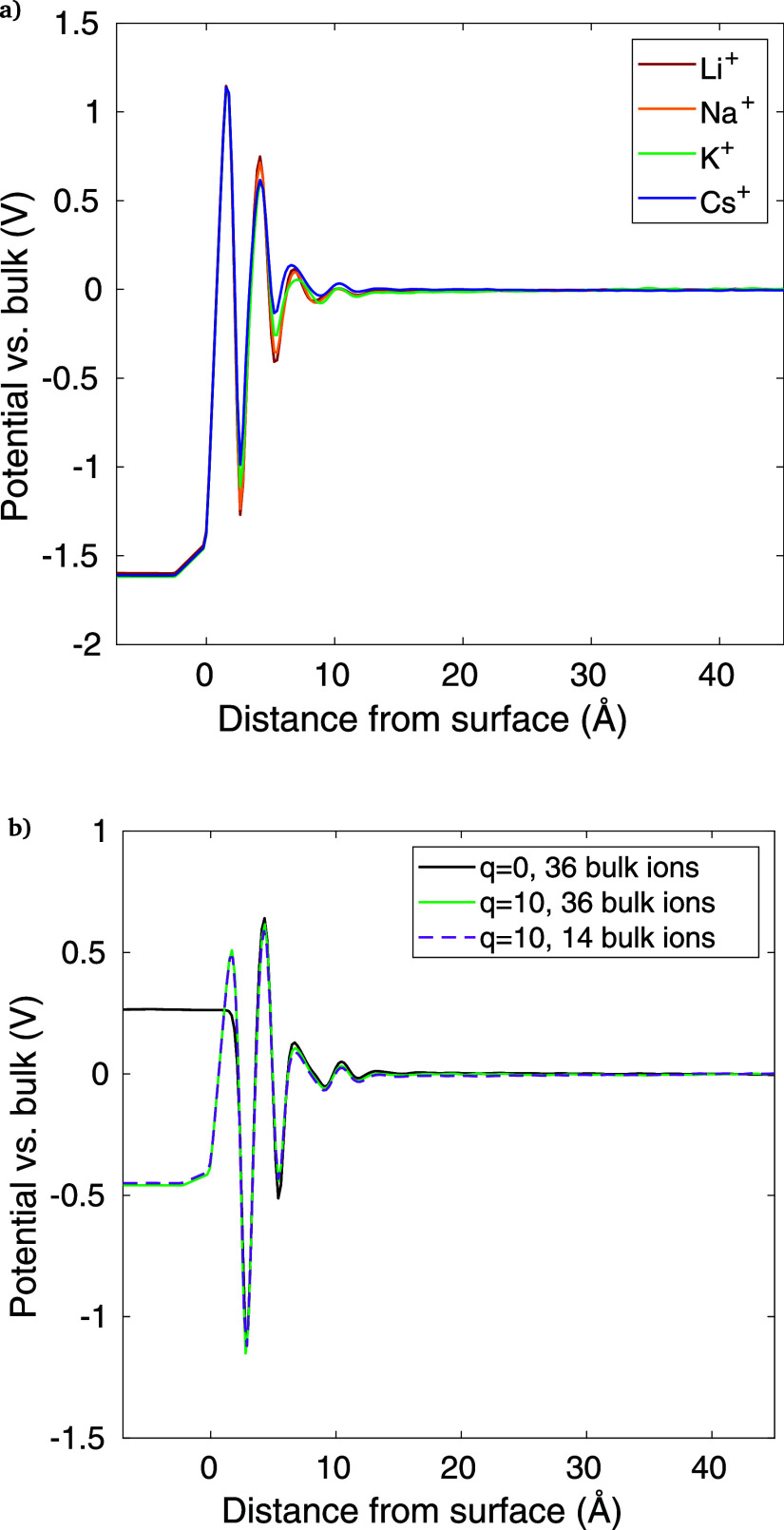
Potential profile in the EDL comparing (a) various cations with *q* = 30 e^–^ and (b) K^+^ with *q* = 10 e^–^, showing bulk ion concentrations of 14 vs 36 (i.e., the entire simulation contains this number of K^+^ and HCO_3_^–^ plus 10 extra K^+^ for each EDL).

Given this line of research, we calculate capacitances in our MD simulations by taking the electrode charge divided by the difference in electrode potential between the simulation and the PZC (*q* = 0) simulation. The electrode potentials are taken as the innermost potential in the electrode (i.e., at *z* = −7 Å). We find the EDL capacitance under three sets of conditions. (A) For the K^+^ simulations with *q* = 10, 20, and 30, we find the capacitances are essentially independent of the applied charge or potential, all falling between 7 and 9 μF cm^–2^. (B) For different cations (potential profiles shown in Figure 9a), the potential profile is very similar for each cation, with an EDL capacitance of 8.2 μF cm^–2^ for all cations. (C) For the *q* = 10 e^–^ case, the number of cations and anions in the bulk is reduced from the original 36 to 14, and the same capacitance (7.2 μF cm^–2^) is found for both concentrations (Figure 9b). Essentially, the capacitance is unchanged under all of these various conditions. Though these values are similar to results from published MD models using the SPC/E water molecule,^53^ they are below the experimental results for electrolytes with high concentration, typically in the range of ∼20 μF cm^–2^ for electrode potentials well below the PZC.^31^

Differential capacitance experiments on Ag^32^ and Pt^44^ often show a peak near the PZC, in direct contrast to the capacitance *minimum* predicted by GCS theory, unless extremely low ionic concentrations are used.^54^ Explaining this peak has recently become a focus of intense research. To explain the trends under low electrolyte concentrations, Schmickler^52^ proposed a specific anion adsorption, while Doblhoff-Dier and Koper^55^ include both an anion- and cation-specific adsorption due to a yet-undetermined attractive force. At high electrolyte concentrations (such as the 0.25 M KHCO_3_ in this study), according to Doblhof-Dier and Koper,^44^ the capacitance peak near the PZC could be attributed to either (a) water reorientation at the solid–electrolyte interface, (b) chemisorption of water, or (c) ion crowding effects. What can MD tell us about these possible explanations? Regarding (c), as discussed in Section 4.5, we do not see any strong evidence of ion crowding effects, which implies that option (c) is unlikely from the MD perspective. Furthermore, in our MD model neither the identity, concentration, nor potential range lead to any observable change in the capacitance, which indicates the peak in capacitance is due to something not captured by MD. This appears to favor the explanation being chemisorption and/or water reorientation. As for the details of (a) and (b), the present MD model is not well-suited to answer these questions. Water reorientation is modeled in MD, but the predicted capacitance being below experimental values indicates the reorientation is likely not captured with sufficient accuracy, at least with the SPC/E water model. Regarding (b), chemisorption of H, OH, or ions^52,55^ is not modeled with classical MD, so this is better studied with AIMD.

## 5 Discussion

One limitation of classical MD is in the accuracy of the force field used, and in this study the parameters were taken from literature. We use SPC/E water because it is one of the most widely used water molecules. In the future, force fields could be parametrized under more relevant conditions to CO_2_R. Furthermore, it would be useful to see if the main trends shown here are also shown by AIMD, which would resolve any questions about the accuracy of the force field parameters employed in the MD model.

Under actual CO_2_R conditions, CO_2_ continuously diffuses from the bulk toward the cathode surface, where it is reduced to CO or other products. One relevant question is if the EDL structure shown here is valid, given that in MD we model no CO_2_ consumption or CO generation. To answer this question, it helps to understand the frequency of reactions within the MD context. Given an electrode with a surface of 54.3 by 57.8 Å, and assuming a CO current density of 10 mA cm^–2^, one CO_2_ molecule would convert to CO every 104,000 ns, very long compared to the 10 ns production run in this study (and requiring 19 years of computation time). Thus, the CO_2_R reaction is exceptionally rare on the length and time scales modeled by MD, and one CO_2_ molecule disappearing infrequently will not affect the results of the EDL structure as long as the concentration outside the EDL is maintained at the desired level, such as the target of 0.034 M in this study. However, when there is an appreciable current density, CO_2_ cannot diffuse from the bulk fast enough to maintain the bulk concentration, so the concentration in the vicinity of the electrode drops to less than the bulk value. Therefore, it may be relevant to model lower CO_2_ concentrations MD, but this was not pursued because the averages become less reliable unless larger/longer simulations are run, becoming computationally unreasonable.

Assuming MD presents the more realistic picture of the EDL than the continuum models, the development of PNP/GMPNP could follow several paths forward. Adding a layer of partially hydrated ions or modifying the size of the hydration shell (following Ringe^10^) could be one way. Another approach would be to switch from PNP/GPMPNP, based on dilute solution theory, to using concentrated solution theory,^25^ which may be more applicable given the high concentrations in the EDL. Coupling MD to continuum simulations may also be a fruitful approach, using the atomic-level detail of MD to model the EDL while relying on the continuum model for the diffusion layer and homogeneous reactions. MD is somewhat computationally costly but still attainable, with each simulation presented here taking roughly 4 days of computation time (54 processors at 3 GHz). Finally, coupling classical MD to AIMD for this situation may be useful to cover the length scales and incorporate chemisorption effects.

## 6 Conclusions

In this study, a direct comparison is made between continuum models for CO_2_R and classical MD simulations. The MD model features a two-cathode setup and constant potential electrodes, a method not previously used in CO_2_R. MD shows cations forming two adsorbed layers, including an inner adsorbed layer with cations shedding some of their hydration shell and an outer layer of fully hydrated cations. The surface density of the inner adsorbed layer increases with more negative applied potentials, and larger cations (Cs^+^) tend to closely adsorb more than smaller ones (Li^+^). In contrast, the continuum models following GCS theory usually assume only a single nonspecifically adsorbed layer at the OHP, a difference that changes the distribution of cations as well as all other modeled species. Potential profiles in MD oscillate until ∼12 Å from the electrode surface, whereas these oscillations are not resolved in continuum models. Furthermore, MD includes the surface interactions from van der Waals forces and image charges between solute ions and the electrode, causing concentration gradients even at the PZC, while in continuum models, there are no concentration gradients at the PZC. Continuum models that include the steric effect predict CO_2_ to be mostly excluded at the cathode surface due to the crowding of cations, yet we find little evidence to support these predictions from the MD results. With the presented MD model, the EDL capacitance is in the range of 7–9 μF cm^–2^, which does not change with the cation identity, bulk concentration, or applied charge. This implies the large changes in capacitance observed experimentally are likely caused by water-electrode interactions, such as chemisorption or water molecule reorientation effects, not captured by the SPC/E water molecule in MD.

Considering these differences in the results between the two methods, it appears the continuum models excel at large length and time scales and where concentrations are low, but they fail to reproduce the trends shown by MD within ∼1 nm of the electrode, where concentrations are high, potentials are driven by polarized water molecules, and electrode–electrolyte interactions become important. Future work may include improving the continuum formulation, coupling between MD and continuum models, and improving upon the MD model parameters to more effectively capture the EDL capacitance.

## Data Availability

Data from plots is available. See DOI: 10.5281/zenodo.8233940.

## Supporting Information Available

Model parameters, symmetric vs asymmetric MD domains, and a comparison of K^+^ force field parameters (PDF)
