# Supplementary material for: Contrasting Views of the Electric Double Layer in Electrochemical CO2 Reduction: Continuum Models vs Molecular Dynamics
Source: J Phys Chem C Nanomater Interfaces. 2024 Jun 14;128(25):10450–64. doi: 10.1021/acs.jpcc.4c03469 (PMC11215773; doi:10.1021/acs.jpcc.4c03469)
Supplement: Supplementary file 1 — jp4c03469_si_001.pdf [file jp4c03469_si_001.pdf]

Contrasting Views of the Electric Double Layer in Electrochemical CO_2 Reduction: Continuum Models vs. Molecular Dynamics

Supplementary Information

Evan Johnson,[a] and Sophia Haussener*[a]

[a]*Laboratory of Renewable Energy Science and Engineering, École Polytechnique Fédérale de Lausanne, Station 9, 1015 Lausanne, Switzerland*

*Phone: +41 21 693 3878; E-mail: sophia.haussener@epfl.ch

S1 Model Parameters

Table S1 Parameters used in GMPNP model.

Parameter	Value	Unit	Ref.
Steric Diameters			
a_{K^+}	0.662	nm	[1]
$a_{HCO_3^-}$	0.800	nm	[1]
a_{CO_2}	0.230	nm	[1]
Diffusivities			
D_{K^+}	1.957E-9	$m^2\ s^{-1}$	[1]
$D_{HCO_3^-}$	1.185E-9	$m^2\ s^{-1}$	[1]
D_{CO_2}	1.910E-9	$m^2\ s^{-1}$	[1]

S2 Atomic Concentrations of HCO_3^- and CO_2

The concentration of the atoms in HCO_3^- and CO_2 are shown in Fig. S1. The concentration of O is divided by 3 in the HCO_3^- plot and by 2 in the CO_2 plot, so concentrations of each atom type can be compared more directly. The HCO_3^- plot shows that H, with a partial charge of +0.4 e^-, tends to be oriented towards the electrode. The positive charge on H likely contributes to the tendency for HCO_3^- ions to stay near the electrode, even though the ion is negatively charged overall. In the plot for CO_2, concentrations of C and O are nearly identical (once dividing the O concentration by 2), meaning CO_2 molecules remain essentially parallel to the surface. This is likely because the C atom carries a +0.7 e^- charge, so the position where CO_2 can be closest and is most attracted to the cathode is when it is parallel to the electrode surface.

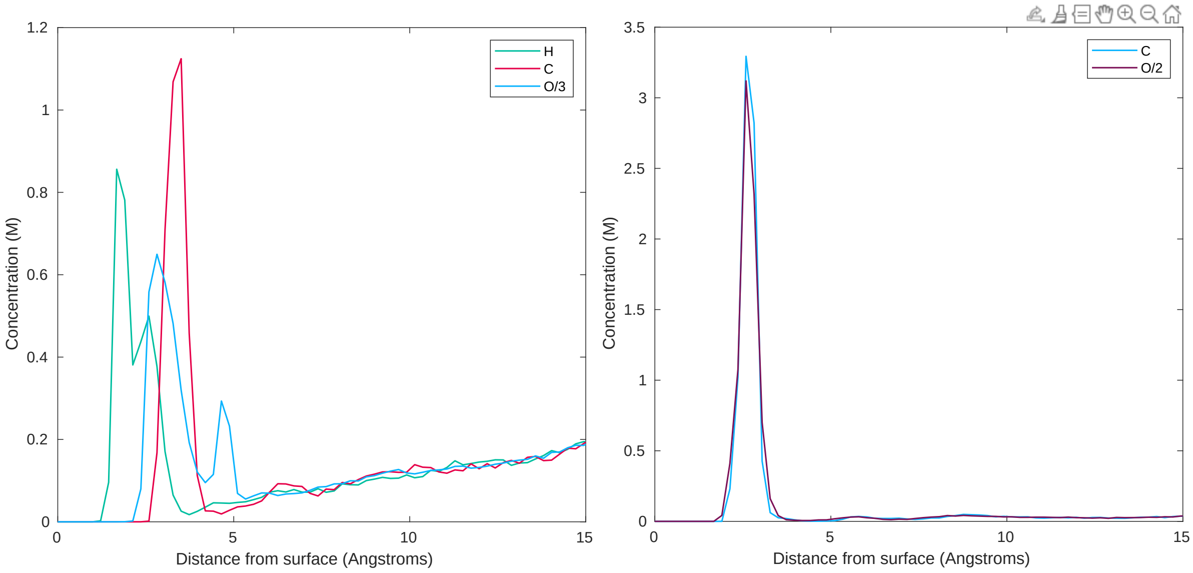

Figure S1 Individual atomic concentrations of (a) HCO_3^- and (b) CO_2, for the q=30 e^- case with K^+ as the cation. Concentration of O is divided by 3 in HCO_3^- and by 2 in CO_2 to better compare concentrations of the constituent atoms.

S3 Symmetric Domain

The symmetric domain with two negatively charged electrodes is chosen over an asymmetrically charged (positive and negative) cell primarily due to computational efficiency. The side of the simulation containing the positively charged electrode is not relevant for CO_2R, so half of the data would have to be immediately be discarded, making the symmetric domain essentially twice as computationally efficient. In the symmetrical cell we expect the EDL formed on each negatively charged electrode to be essentially the same as the negative half of an asymmetric cell. Fig. S2 shows this equivalence. The charge density, electric field, and potential are shown for both types of cell for the "K^+, q=10" case. In the asymmetric cell, the left electrode is specified with q=-10 e^- and the right with q=10 e^-. In the symmetric cell, each electrode is given q=-10 e^-. The left side of the simulations are essentially identical, justifying the choice to use the symmetrical cell.

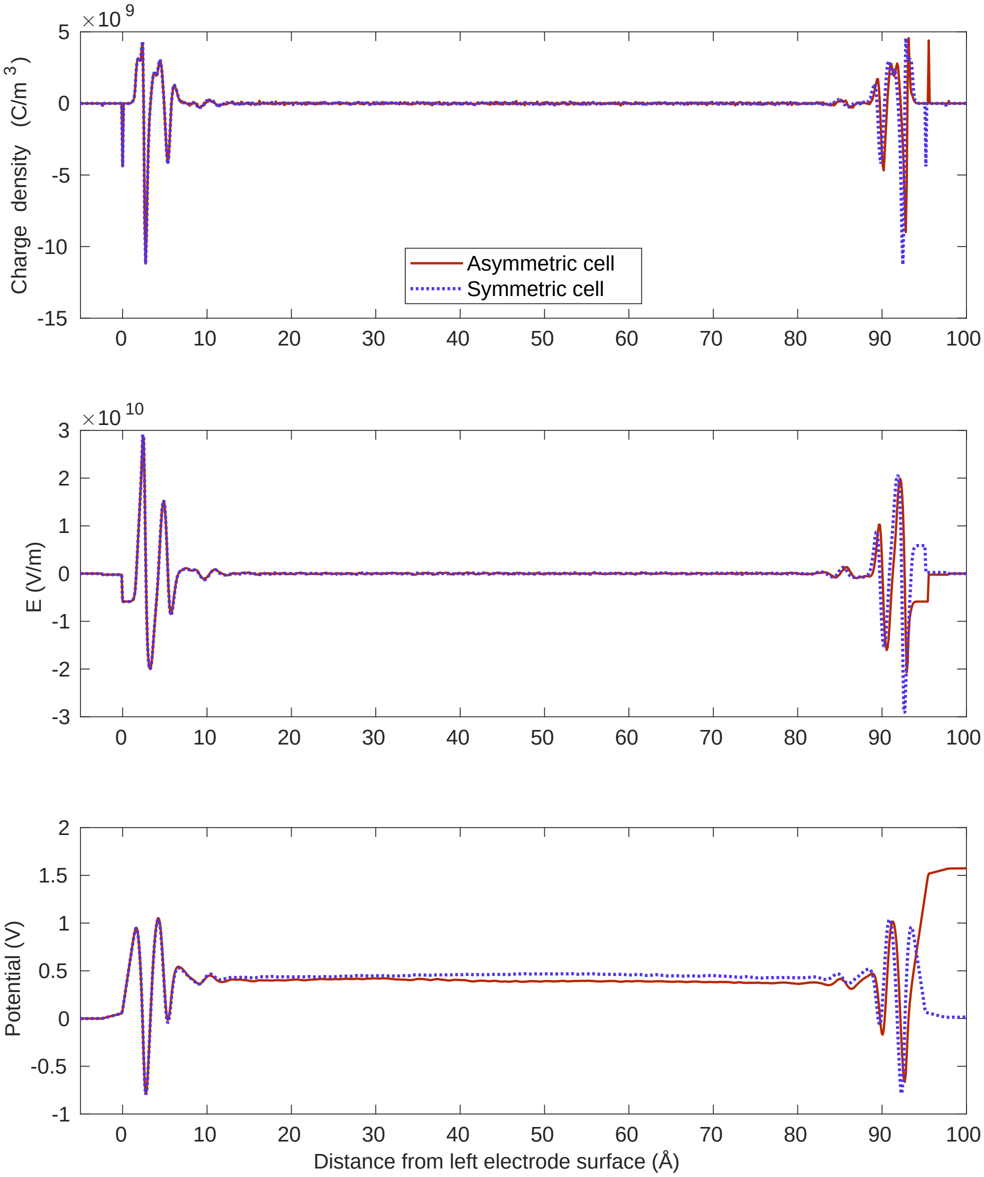

Figure S2 Comparison of symmetric (mirrored simulations, as used throughout this work) and the equivalent asymmetrically charged cell. The symmetric cell has a charge of -10 e^- on each electrode, while the asymmetric cell has -10 e^- on the left electrode and +10 e^- on the right electrode.

S4 Comparison of K+ Force Fields

Several sets of LJ parameters have been used previously in literature, including Jiang[2], Dang[3], and Lee[4]. Both Dang and Lee used the SPC/E water molecule for parameterization, while Jiang used parameters from the CHARMM27 force field. Parameters from Jiang were used for K+ throughout this work, but parameters from Dang and Lee are very similar. Fig. S3 shows MD simulation results using parameters from Jiang and Dang. The K+ profiles are very similar, showing the results are consistent between force fields. We did not model using Lee's K+ parameters, but the parameters are very similar to Jiang and Dang, so results are expected to be very similar as well.

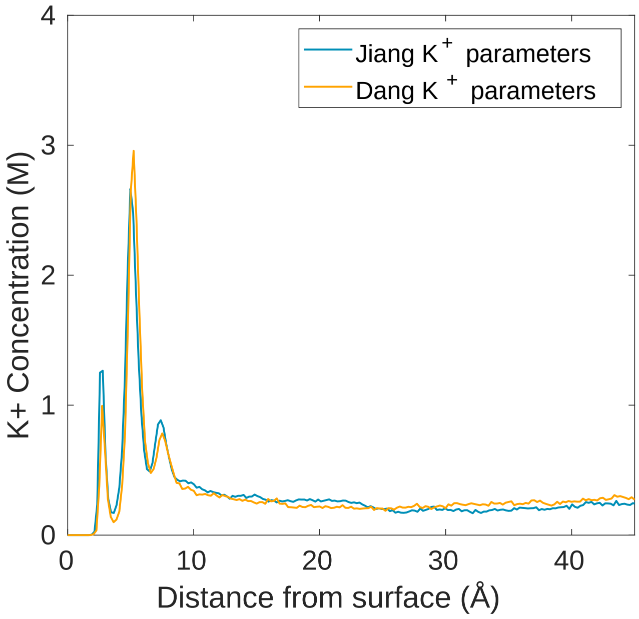

Figure S3 Comparison of results using Lennard-Jones from Jiang[2] and Dang[3].

References

[1] Divya Bohra. *Modeling the carbon dioxide electrocatalysis system*. PhD thesis, Delft University of Technology, 2020.

[2] Gengping Jiang, Chi Cheng, Dan Li, and Jefferson Zhe Liu. Molecular dynamics simulations of the electric double layer capacitance of graphene electrodes in mono-valent aqueous electrolytes. *Nano Research*, 9(1):174–186, January 2016. Company: Springer Distributor: Springer Institution: Springer Label: Springer Number: 1 Publisher: Tsinghua University Press.

[3] Liem X. Dang. Mechanism and Thermodynamics of Ion Selectivity in Aqueous Solutions of 18-Crown-6 Ether: A Molecular Dynamics Study. *Journal of the American Chemical Society*, 117(26):6954–6960, July 1995.

[4] Song Hi Lee and Jayendran C. Rasaiah. Molecular Dynamics Simulation of Ion Mobility. 2. Alkali Metal and Halide Ions Using the SPC/E Model for Water at 25 °C. *The Journal of Physical Chemistry*, 100(4):1420–1425, January 1996. Publisher: American Chemical Society.
